# Human DHEA sulfation requires direct interaction between PAPS synthase 2 and DHEA sulfotransferase SULT2A1

**DOI:** 10.1074/jbc.RA118.002248

**Published:** 2018-05-09

**Authors:** Jonathan W. Mueller, Jan Idkowiak, Tarsis F. Gesteira, Cecilia Vallet, Rebecca Hardman, Johannes van den Boom, Vivek Dhir, Shirley K. Knauer, Edina Rosta, Wiebke Arlt

**Affiliations:** From the ‡ Institute of Metabolism and Systems Research (IMSR), University of Birmingham, Birmingham B15 2TT, United Kingdom,; the § Centre for Endocrinology, Diabetes and Metabolism (CEDAM), Birmingham Health Partners, Birmingham B15 2TH, United Kingdom,; the ¶ Department of Chemistry, King's College London, London SE1 1DB, United Kingdom, and; the Departments of ‖ Molecular Biology II, Centre for Medical Biotechnology (ZMB) and; ** Molecular Biology I, Centre for Medical Biotechnology (ZMB), University of Duisburg-Essen, 45141 Essen, Germany

**Keywords:** steroid hormone, sulfotransferase, molecular docking, enzyme kinetics, protein–protein interaction, dehydroepiandrosterone, DHEAS, PAPS synthase, steroid sulfation pathway

## Abstract

The high-energy sulfate donor 3′-phosphoadenosine-5′-phosphosulfate (PAPS), generated by human PAPS synthase isoforms PAPSS1 and PAPSS2, is required for all human sulfation pathways. Sulfotransferase SULT2A1 uses PAPS for sulfation of the androgen precursor dehydroepiandrosterone (DHEA), thereby reducing downstream activation of DHEA to active androgens. Human *PAPSS2* mutations manifest with undetectable DHEA sulfate, androgen excess, and metabolic disease, suggesting that ubiquitous PAPSS1 cannot compensate for deficient PAPSS2 in supporting DHEA sulfation. In knockdown studies in human adrenocortical NCI-H295R1 cells, we found that PAPSS2, but not PAPSS1, is required for efficient DHEA sulfation. Specific APS kinase activity, the rate-limiting step in PAPS biosynthesis, did not differ between PAPSS1 and PAPSS2. Co-expression of cytoplasmic SULT2A1 with a cytoplasmic PAPSS2 variant supported DHEA sulfation more efficiently than co-expression with nuclear PAPSS2 or nuclear/cytosolic PAPSS1. Proximity ligation assays revealed protein–protein interactions between SULT2A1 and PAPSS2 and, to a lesser extent, PAPSS1. Molecular docking studies showed a putative binding site for SULT2A1 within the PAPSS2 APS kinase domain. Energy-dependent scoring of docking solutions identified the interaction as specific for the PAPSS2 and SULT2A1 isoforms. These findings elucidate the mechanistic basis for the selective requirement for PAPSS2 in human DHEA sulfation.

## Introduction

Sulfation pathways are a vital part of human physiology, encompassing the central triad of sulfate activation, transfer, and removal (1, 2). Many sulfotransferases ensure substrate specificity of sulfation; the 62 human sulfotransferase genes only have 46 direct counterparts in the mouse genome (2, 3). In contrast, 3′-phosphoadenosine-5′-phosphosulfate (PAPS)^2^ synthases, the enzymes responsible for sulfate activation, are represented by only two genes in humans, *PAPSS1* and *PAPSS2* (4). This gene pair is evolutionary conserved in all vertebrate genomes investigated so far; RNA splice forms and additional teleost-specific gene duplications of *PAPSS2* are the only exceptions (4).

The primary role of PAPS synthases is to provide the many and diverse sulfotransferases with the high-energy sulfate donor PAPS. PAPS availability is generally the limiting factor in this system (5), due to the high energetic cost of PAPS biosynthesis. First, nucleophilic sulfate needs to attack the α-phosphorus of ATP, catalyzed by ATP sulfurylase, resulting in the formation of APS (adenosine 5′-phosphosulfate) and the release of pyrophosphate (6). This reaction lies heavily on the educt side, with an equilibrium constant for APS formation of 10^−8^ (7); a fact exploited in pyrosequencing (8). To pull the reaction toward product formation, pyrophosphatases swiftly cleave the pyrophosphate and APS kinase phosphorylates APS at its ribose 3′ position, resulting in the formation of PAPS (6). Once PAPS is used in sulfation reactions, the remaining bis-phosphorylated nucleotide PAP (3′-phosphoadenosine-5′-phosphate) needs to be cleaved by dedicated PAP phosphatases (9, 10). In animal genomes, ATP sulfurylase and APS kinase are fused to the above mentioned bifunctional PAPS synthases (4, 11). The phosphorylation of APS by APS kinase is regarded the rate-limiting step of overall PAPS biosynthesis (6, 12).

PAPS synthases 1 and 2 are very similar enzyme isoforms with 78% amino acid identity (4), but with an unknown degree of functional overlap. The clinical phenotype of human loss-of-function mutations in the gene encoding PAPSS2 have suggested differential roles for PAPSS1 and PAPSS2 in human sulfation pathways (2, 13, 14). Inactivating *PAPSS2* mutations have been reported to present with skeletal malformations, specifically variable phenotypes of spondyloepimetaphyseal dysplasia (14, 15), and with biochemical and clinical evidence of androgen excess (13, 14). Sulfation of the androgen precursor dehydroepiandrosterone (DHEA) reduces the availability of nonsulfated DHEA for downstream conversion to androgens. Hence, impaired DHEA sulfation results in a higher rate of androgen activation and clinically in androgen excess phenotypes such as polycystic ovary syndrome (13, 14, 16). Crucially, the phenotype of human *PAPSS2* deficiency proves that with regard to DHEA sulfation and bone and chondrocyte development, human *PAPSS1*, the gene encoding the only other PAPS synthase, appears not to be able to compensate for the loss of *PAPSS2* gene function.

There has been considerable debate about the cause of the divergent functions of the two PAPS synthase isoforms. Differences in subcellular localization have been suggested as an underlying mechanism, with PAPSS1 reported as nuclear protein and PAPSS2 as primarily located in the cytoplasm (17). However, this was recognized as oversimplification as both enzymes actively shuttle between nucleus and cytoplasm, guided by conserved nuclear localization and nuclear export signals (18). Another aspect was an apparent difference in specific enzymatic activity. When assayed as pseudo one-step enzymes, PAPSS2 was reported to display a much higher *k*_cat_/*K_m_* value than PAPSS1 (19). When assaying only the rate-limiting step of overall PAPS biosynthesis, the APS kinase-catalyzed reaction, this difference was no longer observed (20). Differences in tissue-specific distribution of the two PAPS synthases have also been reported (13, 19, 21). Nevertheless, none of those studies could sufficiently explain why the *PAPSS1* gene cannot compensate for the loss of PAPSS2 due to inactivating *PAPSS2* mutations.

**Figure 1.**
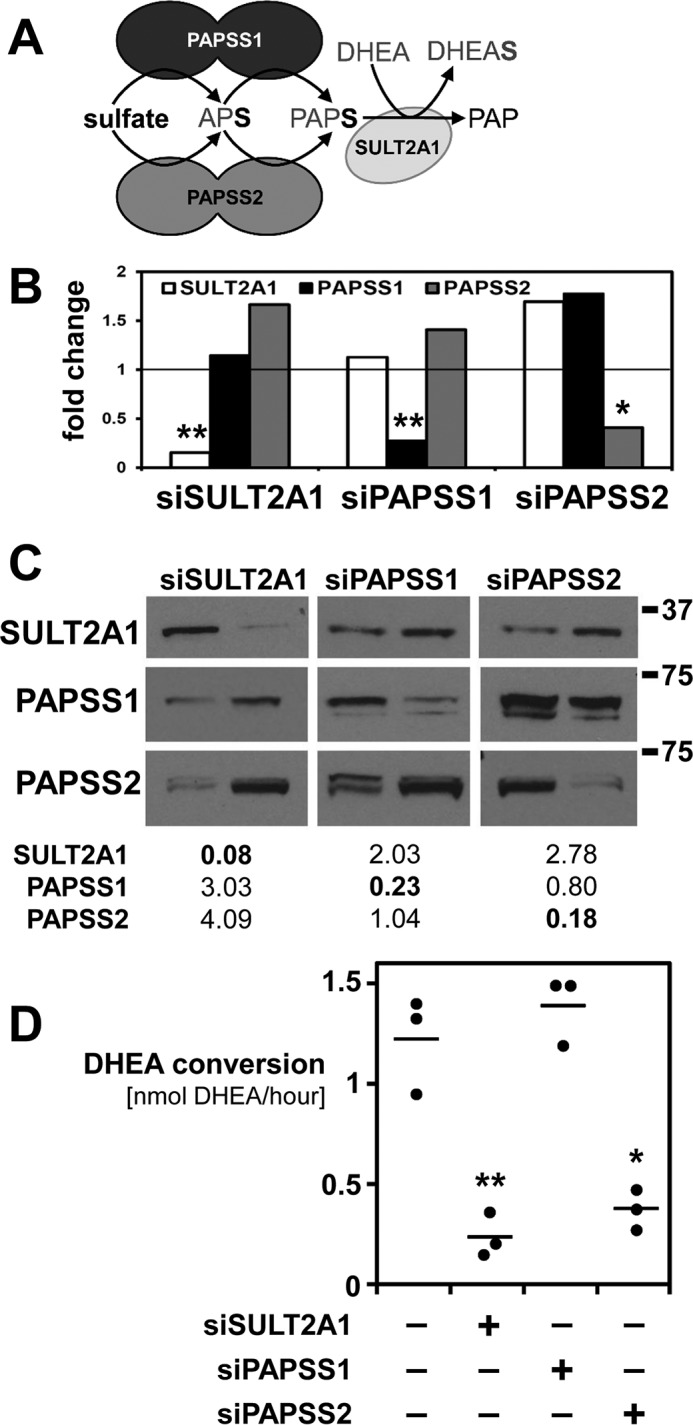
**Knockdown of components of the DHEA sulfation pathway.** *A*, schematic representation of the DHEA sulfation pathway. Activated sulfate in the form of PAPS is produced by either PAPSS1 or PAPSS2 and then used by the sulfotransferase SULT2A1 to convert DHEA to DHEAS. *B* and *C*, siRNA-mediated knockdown of SULT2A1, PAPSS1, or PAPSS2 in adrenocortical NCI-H295R1 cells was verified by real-time PCR and Western blotting. A scrambled oligonucleotide served as control (*ctrl*). Real-time PCR data normalized to 18S rRNA, fold-change relative to that control. Densitometric quantification of Western blots revealed knockdown efficiencies of up to 90% on the protein level. Double bands were interpreted as degradation products and jointly analyzed. *D*, DHEA sulfation was assayed for all knockdowns mentioned above, revealing functional differences between PAPSS1 and PAPSS2 for DHEA sulfation by sulfotransferase SULT2A1. Three biological replicates and their average are shown; each *dot* consists of at least three technical replicates. Normally distributed data were analyzed by one-way ANOVA (*p* value < 0.001) and post-hoc Bonferroni tests (*, *p* < 0.05; **, *p* < 0.01) relative to the control.

Here we report experimental evidence for nonoverlapping functionality of PAPSS1 and PAPSS2 with regard to DHEA sulfation by SULT2A1 (Fig. 1*A*). Proximity ligation assays (PLAs) detect a novel protein–protein interaction between SULT2A1 and PAPSS2 and molecular docking suggests SULT2A1 contacts the APS kinase domain of PAPSS2. This newly described protein–protein interaction, specific to the PAPSS2 and SULT2A1 isoforms, may guide the directionality of sulfation pathways in tissues with equal expression of PAPS synthases and high expression levels of various sulfotransferases.

## Results

### PAPSS2 is functionally required for DHEA sulfation in a human adrenocortical cell line

The adrenal cortex is a major site of DHEA sulfation. We used human adrenocortical NCI-H295R1 cells, which we found to express high levels of SULT2A1 mRNA (Δ*C_T_* value 13.4 ± 1.3 relative to 18S rRNA, ± S.D., see also Table S1) and almost identical mRNA levels of PAPSS1 and PAPSS2 (Δ*C_T_* values 16.1 ± 0.8 and 15.8 ± 0.9, respectively). We separately targeted SULT2A1 and both PAPS synthase isoforms by siRNA-mediated knockdown, achieving knockdown efficiencies of up to 90% at mRNA (Fig. 1*B*) and protein levels (Fig. 1*C*). We then carried out functional assays and found that siRNA-mediated knockdown of SULT2A1 and PAPSS2 reduced DHEA sulfation rates to 19 and 30%, respectively, whereas depletion of PAPSS1 showed no discernable effect on DHEA sulfation (Fig. 1*D*). This suggests nonoverlapping functionality of the two human PAPS synthase proteins with regard to DHEA sulfation.

### Enzymatic properties are very similar for both PAPS synthases

**Figure 2.**
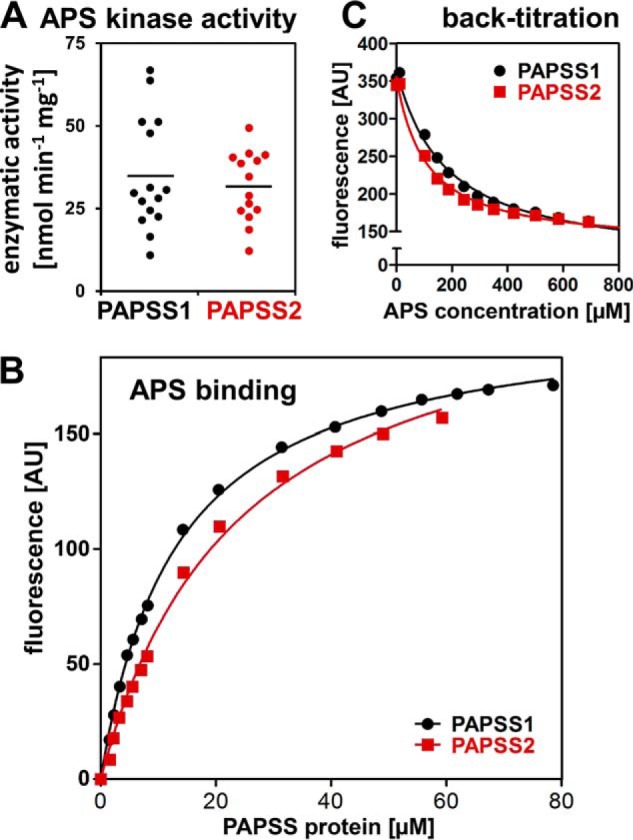
**APS kinase activity and APS binding properties of human PAPS synthases.** *A*, APS kinase activity was measured in a coupled enzymatic assay where ADP production is linked to NADH consumption via pyruvate kinase and lactate dehydrogenase. 15 individual velocity measurements from two different batches are shown. Please refer to Table 1 for the averaged specific activity. *B*, APS binding studies where fluorescently labeled APS (1 μm mant-APS) was titrated with increasing concentrations of PAPS synthase protein. Data were fitted assuming one binding site. *C*, back-titration of 1 μm mant-APS and 50 μm PAPSS protein with increasing concentrations of APS. As mant-APS can be displaced by APS, the fluorescent mant moiety did not interfere with binding to the protein.

To determine whether different enzyme activities may explain the above described differences in functionality, we determined specific APS kinase activities of recombinant PAPSS1 and PAPSS2 proteins in a coupled spectrophotometric assay. APS kinase activity is known to be the rate-limiting step of overall PAPS biosynthesis (12). Using two different enzyme batches, performing multiple repeat measurements (Fig. 2*A*), specific APS kinase activities appeared nondistinguishable, 34.9 ± 17.0 nmol min^−1^ mg^−1^ for PAPSS1 and 31.6 ± 10.6 nmol min^−1^ mg^−1^ for PAPSS2 (Table 1), in line with previous findings (20).

**Table 1:**
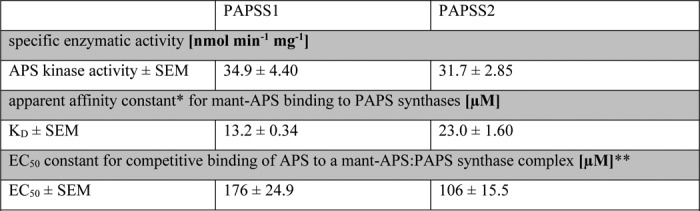
**Enzymatic characterization of human PAPS synthase isoforms**

* Assuming a single nucleotide binding site.

** EC_50_ was measured in the presence of 50 μM PAPSS protein and 1 μM mant-APS.

The nucleotide APS has been reported to be a highly effective modulator of PAPS synthase proteins (4, 6). Hence, we determined binding affinity (*K_D_*) of a fluorescently labeled APS derivative (mant-APS) to recombinant PAPS synthase proteins (Fig. 2*B*). Theoretically, APS could bind to all six nucleotide-binding sites of dimeric PAPS synthase proteins (6); but fitting to the Hill equation resulted in very weak cooperativity with Hill coefficients close to 1 (1.09 ± 0.02 and 1.22 ± 0.05 for PAPSS1 and PAPSS2, respectively). Hence, data were fitted assuming one binding site, resulting in apparent *K_D_* values of 13.2 ± 0.3 and 23.0 ± 1.6 μm for PAPSS1 and PAPSS2, respectively. Titrations of preformed PAPS synthase–mant-APS complexes with APS confirmed that both nucleotides actually bound to the same site(s) within the enzyme (Fig. 2*C*). We also attempted to determine ATP sulfurylase activity; however, this was hindered by considerable batch-to-batch variability in PAPSS2 (data not shown). Without any significant differences in the APS kinase activity of PAPSS1 and PAPSS2 and very similar affinities for the modulating APS nucleotide, we conclude that differences in enzymatic activity cannot explain the above described functional differences.

### Cytosmic PAPSS2 supports SULT2A1 activity

**Figure 3.**
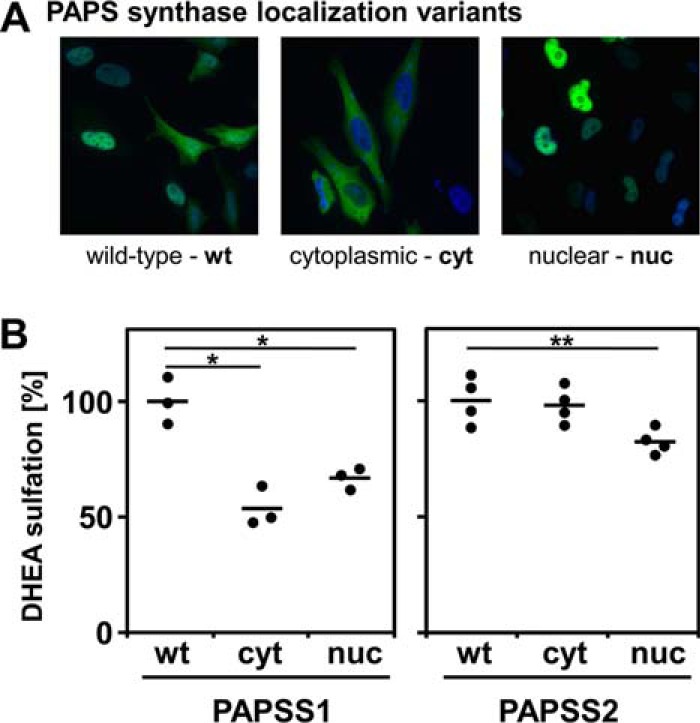
**Cytoplasmic PAPSS2 best supports cytoplasmic SULT2A1 activity.** *A*, HEK293 cells were transfected with cytoplasmic sulfotransferase SULT2A1 as well as cytoplasmic (PAPSS1 K9A,K10A and PAPSS2 K6A,K8A) or nuclear protein variants of PAPS synthases (PAPSS1 R111A,R112A and PAPSS2 R101A,R102A) as EGFP fusion proteins. The different variants for PAPSS1 are shown exemplarily; their localization was as described before (18). ×600 magnification. *B*, DHEA sulfation was assayed for these different PAPS synthase variants. Each point represents the average from triplicate measurements. Normally distributed data were analyzed by one-way ANOVA (*p* value < 0.001) and post-hoc Bonferroni tests (*, *p* < 0.05; **, *p* < 0.01) relative to the control.

To determine whether different cellular localization of PAPS synthases might contribute to their functional differences, we examined subcellular localization variants of PAPS synthases 1 and 2 with preferential nuclear or cytoplasmic localization (18) for their ability to support DHEA sulfation by SULT2A1 (Fig. 3*A*). A nonsteroidogenic HEK293 cellular background with no notable expression of these sulfation enzymes was used to co-express PAPS synthase protein variants with cytoplasmic SULT2A1; activity of this sulfation pathway was then tested in DHEA sulfation assays (Fig. 3*B*). Cytoplasmic PAPSS1 expression only supported DHEA sulfation to 61% of WT protein activity (conversion rates in (nmol DHEA/h) are given in Table S2). Both nuclear PAPS synthases showed less DHEA sulfation than the respective WT protein (72 and 82% of WT for PAPSS1 and PAPSS2, respectively). Only cytoplasmic PAPSS2 was as effective in supporting DHEA sulfation as WT PAPSS2 (Fig. 3*B*). This effect of subcellular location on the ability of PAPSS2 to support DHEA sulfation by SULT2A1 led us to hypothesize that these sulfation pathway proteins might physically interact.

### Proximity ligation assays detect a protein–protein interaction of PAPSS2 and SULT2A1

**Figure 4.**
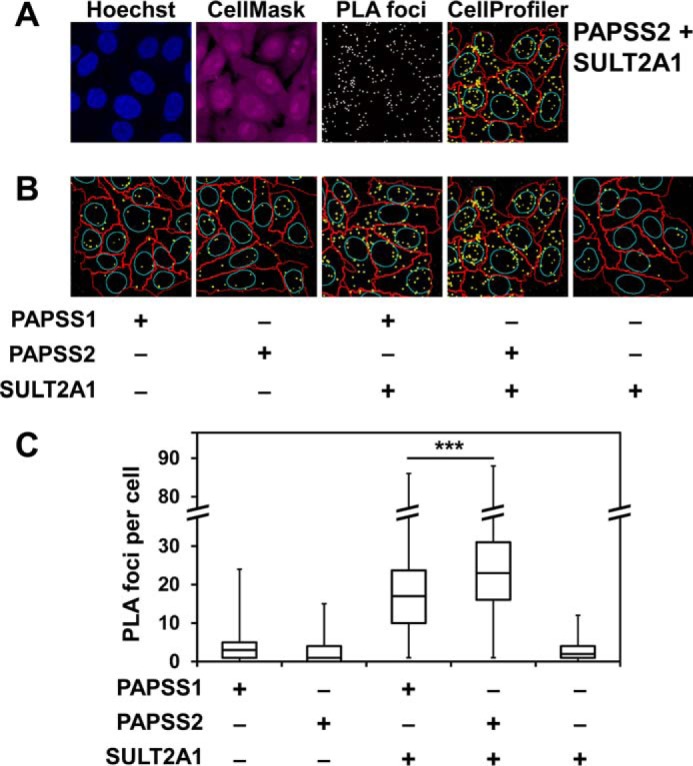
**A physical interaction of PAPSS2 and SULT2A1 detected by proximity ligation assays.** *A*, representative images of the proximity ligation assay between PAPSS2 and SULT2A1 as well as subsequent analysis with CellProfiler. Endogenous PAPS synthases and SULT2A1 were detected by mouse monoclonal antibodies for PAPSS1 or PAPSS2 and a rabbit SULT2A1 polyclonal antibody in a HepG2 cell line. PLA analysis, including automated cell and nucleus recognition and foci counting was carried out using CellProfiler software. Cell nuclei were stained with Hoechst 33342 (*blue*); CellMask staining is shown in *magenta*. PLA foci are shown in *white* in the single channel picture. In the output image of CellProfiler analysis edges of nuclei are represented in *cyan*, cell boarders in *red*, and PLA foci in *yellow. B*, CellProfiler results for all other combinations. Negative controls were generated using only one primary antibody at a time. ×600 magnification for *A* and *B. C*, box-and-whisker analysis of the PLA foci number per cell from at least 400 cells pooled from three independent experiments. Data were found to be not normally distributed; hence, one-way ANOVA (*p* value < 0.001) and post hoc Bonferroni tests (***, *p* < 0.001) were performed after data were square root transformed.

To test for a physical interaction between PAPSS2 and SULT2A1, we employed PLA technology after demonstrating that this putative interaction was not amenable to detection by GFP-trap pulldown (Fig. S1*A*). Hypothesizing that the putative PAPSS2–SULT2A1 interaction is of transient character, we employed PLA technology, which is well-suited to capture transient interactions (22). DNA-linked secondary antibodies and a linker oligo enable rolling circle amplification and signal generation (“foci”) only if the primary antibodies against SULT2A1 and PAPSS1/PAPSS2 have bound less than 40 nm apart. One “focus” is assumed to correspond to one ligation event and the average number of “foci per cell” is interpreted as binding strength (23). PLA technology combined with automated cell, nucleus, and foci recognition (Fig. 4*A*) allows for the analysis of large numbers of cells per staining, more than 400 cells per condition in our analysis. Foci per cell were clearly elevated in the staining for PAPSS2 and SULT2A1, indicative of a physical interaction between these proteins (Fig. 4*B*). Furthermore, foci per cell were significantly higher for PAPSS2–SULT2A1 than for a corresponding staining of SULT2A1 and PAPSS1 (Fig. 4*C*).

### SULT2A1 docks uniformly to the APS kinase domain of PAPSS2

**Figure 5.**
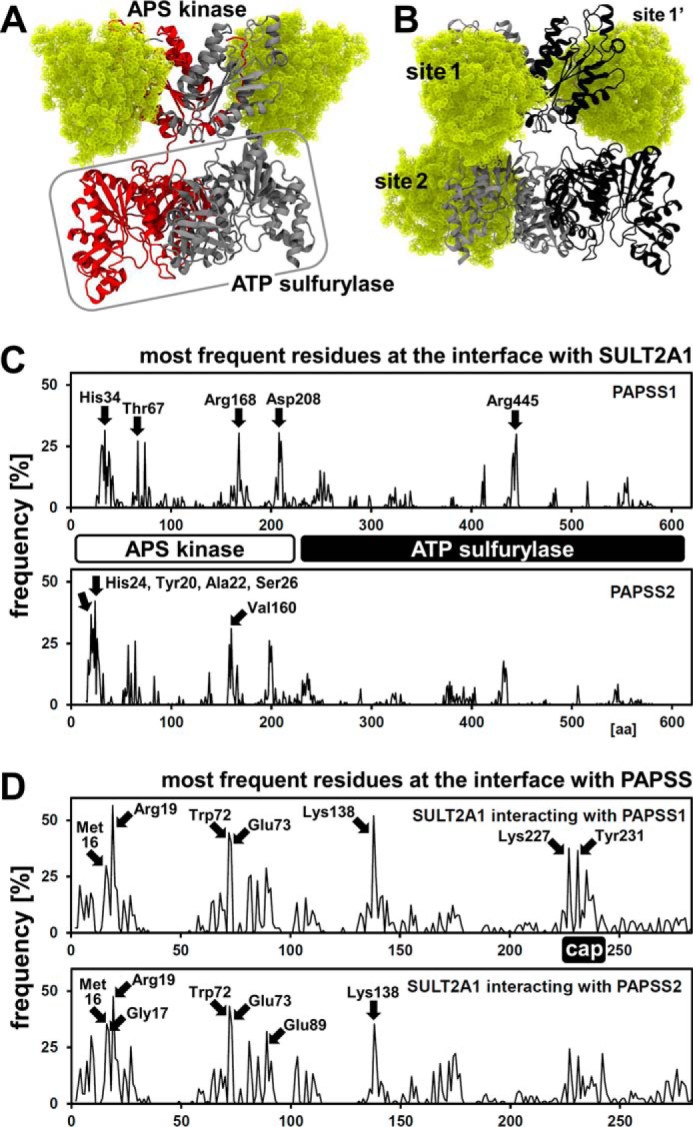
**SULT2A1 docks to the APS kinase domain of PAPSS2.** ClusPro computational docking of three different SULT2A1 crystal structures (PDB codes 1EFH, 3F3Y, and 4IFB) to structural models of PAPSS1 and PAPSS2. *A*, PAPSS2–SULT2A1-docked complexes are shown. PAPSS2 APS kinase is labeled; ATP sulfurylase is labeled and *boxed*. The two PAPSS2 dimeric subunits are *gray* and *red*. Two SULT2A1 molecules are depicted in *yellow*, ball representation; contacting PAPSS2 at its APS kinase domain, sites 1 and 1′. *B*, corresponding representation of PAPSS1–SULT2A1 docking experiments. In addition to sites 1 and 1′, SULT2A1 contacts PAPSS1 also at the ATP sulfurylase domain. Color coding as in *A*, except the two PAPSS1 dimeric subunits, which are *gray* and *black. C* and *D*, statistical analysis of all ClusPro docking experiments, looking from the PAPS synthase side (*C*) and from the SULT2A1 side (*D*). Frequency of individual residues within 3 Å of the other protein was analyzed for PDB 1EFH, 3F3Y, and 4IFB structures separately (30 dockings each) and then averaged. Note the higher number of frequent protein contacts within the APS kinase domain of PAPSS2, compared with the one from PAPSS1. SULT2A1 contacted PAPS synthases mainly via its isoform-specific substrate binding loops; one of these is regarded as “cap.”

To examine the newly detected PAPSS–SULT2A1 complex on a molecular level, we used different available crystal structures of human SULT2A1 and structural information about PAPS synthases for protein–protein docking using ClusPro (24). This procedure revealed a novel protein–interaction interface at the APS kinase domain of PAPSS2, where SULT2A1 binds (Fig. 5*A*). Considering the almost perfect C2 symmetry of the APS kinase domain, we regarded two binding sites as equivalent. Docking SULT2A1 to PAPSS1 resulted in more diffuse complexes, as SULT2A1 populated additional binding sites at the ATP sulfurylase of PAPSS1 (Fig. 5*B*). Statistical analysis of the ensembles of docked complexes confirm this observation (Fig. 5*C*). For PAPSS2, all amino acids found most often at the interface with SULT2A1 cluster within the APS kinase domain, whereas interacting amino acids of PAPSS1 seem to be scattered over the entire protein (Fig. 5*C*). Looking from the SULT2A1 side, the mode of binding to PAPSS1 and PAPSS2 appears to be very similar, involving the substrate-binding loops. One notable difference is that amino acids from the cap, the major substrate-binding loop, are involved in binding to PAPSS1, whereas these amino acids do not play a role in binding to PAPSS2 (Fig. 5*D*).

### The novel protein interaction may be specific for PAPS synthase 2 and the sulfotransferase SULT2A1 from hominids

**Figure 6.**
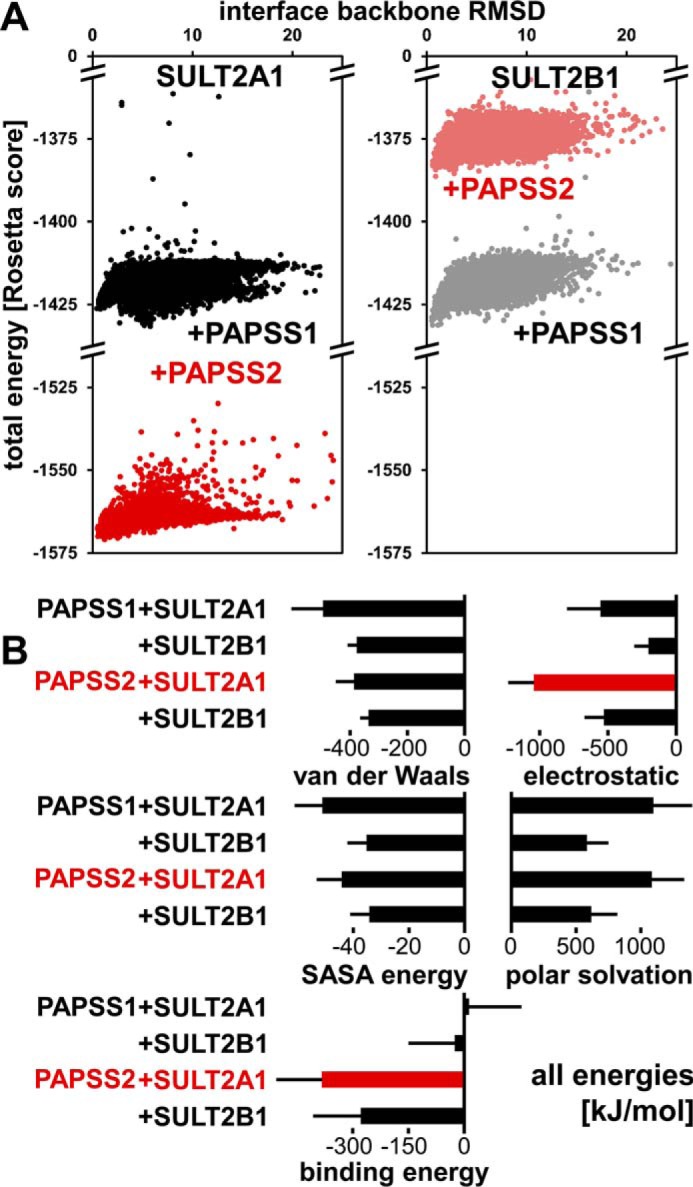
**The PAPSS2–SULT2A1 interaction may be isoform-specific.** *A*, SULT2B1 was selected as homologous sulfotransferase to analyze specificity of the novel PAPSS2–sulfotransferase interaction. PAPS synthase–sulfurylase docking was refined using RosettaDock. At least 10,000 docking experiments are shown where the docking score was correlated with the interface r.m.s. deviation value compared with the average complex. *B*, best solutions from Rosetta were subjected to MD simulations (3 × 20 ns, see Fig. S2 for averaged traces); MM-PBSA energies were derived therefrom, expressed as average ± S.D. from three independent calculations.

Best scoring complexes were then subjected to local docking and re-scoring using RosettaDock (25, 26). The resulting complexes are described by their structural similarity to an average complex (interface r.m.s. deviation) and a docking score representing an energy term (Fig. 6*A*). Docking of PAPSS2 and SULT2A1 resulted in a cloud of docking experiments with a clearly visible funnel toward low r.m.s. deviations and low Rosetta energies (Fig. 6*A*). The corresponding PAPSS1/SULT2A1 docking neither showed such a trend nor was it characterized by similarly favorable Rosetta scores (Fig. 6*A*).

To explore isoform specificity also for the sulfotransferase, we repeated the entire docking procedure with the sulfotransferase SULT2B1 most closely related to SULT2A1 (51% amino acid identity). Although PAPSS1 docking gave similar ensembles both for SULT2A1 and SULT2B1, the PAPSS2–SULT2B1 pairing only gave nonpreferable scores (Fig. 6*A*). Best-scoring complexes from each of these combinations were subjected to molecular dynamics simulations. R.m.s. deviation trajectories showed that all these complexes are stable and converge toward an equilibrium state within the simulation time window (Fig. S2), indicating that energy calculations were appropriate. Free energy MM-PBSA calculations for all four complexes are shown in Fig. 6*B*, van der Waals energies, polar solvation, and SASA energy terms are roughly the same for all four combinations. However, an electrostatics term of −1040 kJ/mol for PAPSS2–SULT2A1 shows that electrostatics favor this interaction; this energy term is about twice as high as those for PAPSS1–SULT2A1 and PAPSS2–SULT2B1; PAPSS1–SULT2B1 is characterized by an even smaller electrostatics term (Fig. 6*B*). Binding energies strongly favor PAPSS2 interactions over PAPSS1 interactions, with both sulfotransferases (Fig. 6*B*). Taken together, the interaction of PAPSS2 and SULT2A1 is mainly driven by electrostatic and entropic binding energy terms.

**Figure 7.**
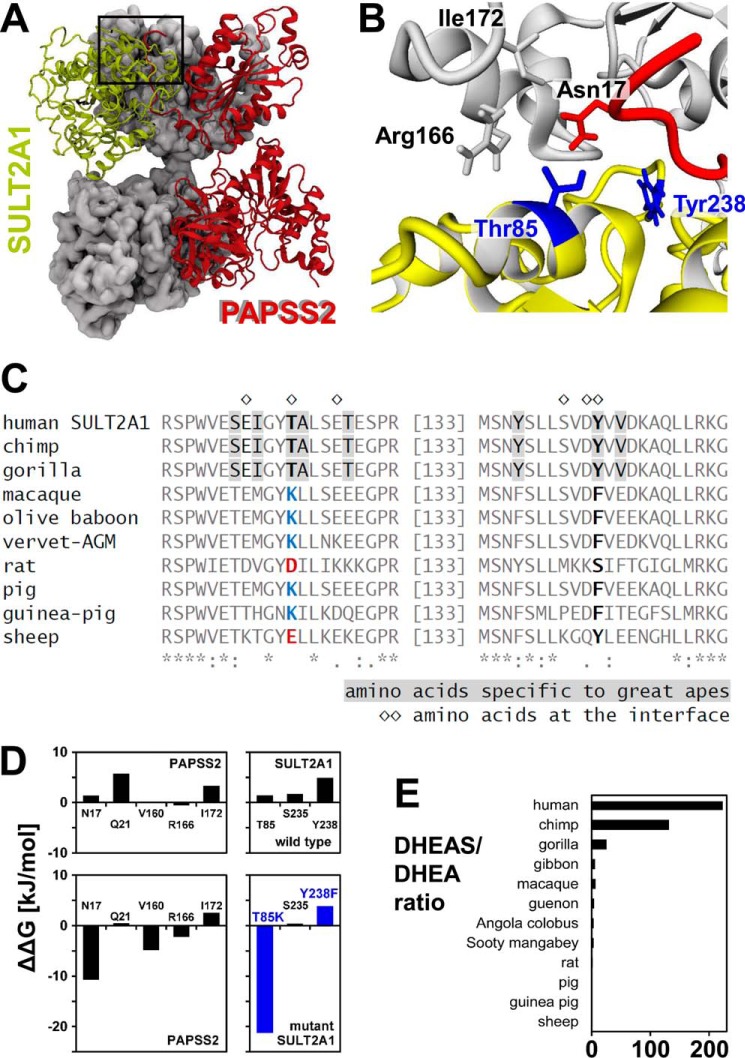
**A PAPSS2–SULT2A1 protein interaction facilitates DHEA sulfation.** *A* and *B*, molecular representation of a PAPSS2–SULT2A1 complex averaged over 15 ns of MD time. The dimeric PAPSS2 subunit proximal to SULT2A1 is depicted in *gray color* and with molecular surface representation; the distant PAPSS2 subunit in *red color* and ribbon representation. SULT2A1 is drawn in *yellow*. Please note the composite nature of the PAPSS2-binding site. Amino acids on the interface are shown in stick representation of the side chains and labeled accordingly. *C*, all SULT2A1 amino acids on the interface with PAPSS2 were highlighted in an alignment of diverse mammalian SULT2A1 protein sequences. The only two interface amino acids that were specific to great apes are Thr^85^ and Tyr^238^ (depicted in *blue* in *B*). *D*, the PAPSS2–SULT2A1 interface was analyzed using Rosetta-based alanine scanning (27). Furthermore, the two great ape-specific amino acids were mutated to their nonhominoid counterparts. T85K resulted in a dramatic loss of stability of the complex. *E*, the hominid-specific PAPSS2–SULT2A1 complex coincides with a higher DHEAS/DHEA ratio in gorilla, chimpanzee, and human. DHEAS/DHEA ratios are derived from Refs. 28 and 29.

An averaged the PAPSS2–SULT2A1 complex is shown in Fig. 7*A*. An important feature is the composite nature of the PAPSS2-binding site, both subunits contribute to the interaction interface (Fig. 7*B*). To determine how general this interaction might be, we looked at all interface amino acids of SULT2A1 within an alignment of various mammalian SULT2A1 species (Fig. 7*C*). There we found only two interface amino acids to be specific to great apes, Thr^85^ and Tyr^238^. These amino acids were then mutated and the effects of these mutations were assessed by Rosetta-based alanine scanning (27). Although the Y238F mutation only moderately compromised the stability of the PAPSS2–SULT2A1 complex, the T85K mutant destabilized the complex by more than 22 kJ/mol (Fig. 7*D*). Furthermore, the double mutation also induced secondary destabilizing effects at Asn^17^ (12 kJ/mol) as well as Arg^166^ and Ile^172^ (5 kJ/mol each). We concluded that the PAPSS2–SULT2A1 interaction was facilitated by amino acid exchanges that only occurred in hominids. It thus correlates with the higher DHEAS/DHEA ratios found in gorilla, chimpanzee, and human (28), but not in other nonhominid primates or other mammals (29, 30).

## Discussion

Our knockdown studies of sulfation pathway enzymes in human adrenal NCI-H295R1 cells, an established model of the adrenal zona reticularis, the main site of DHEA sulfation by SULT2A1, provide experimental evidence for a functional difference of PAPS synthases in the DHEA sulfation pathway. PAPSS2 seems to be better able to support the sulfotransferase SULT2A1 than its enzyme ortholog PAPSS1. Deviations in catalytic properties or subcellular localization are not sufficient to explain the leading role of PAPSS2 in this sulfation pathway. By employing PLAs, we could detect a transient protein–protein interaction between PAPS synthases and SULT2A1. The average number of foci per cell was significantly higher for PAPSS2 than for PAPSS1, indicative of a stronger interaction. Furthermore, molecular docking suggested a specific interaction of SULT2A1 with the APS kinase domain of PAPSS2, whereas analogous docking studies with PAPSS1 suggested a more diffuse interaction pattern. This transient protein–protein interaction provides the mechanistic basis for the observed functional differences between PAPSS1 and PAPSS2 with regard to sulfation of DHEA by SULT2A1, a critical step in controlling biosynthesis of active androgens in humans.

The protein–protein interaction described here is a novel regulatory mechanism to confer directionality to sulfation pathways (Fig. 7). This might be of most relevance in tissues where both PAPS synthases are present at roughly similar levels and where SULT2A1 is co-expressed with other cytoplasmic sulfotransferases. The first condition is met in the adrenocortical NCI-H295R1 cell line of which we started, assuming our real-time Δ*C_T_* values roughly correlate with protein levels, we had nearly identical PAPSS1 and PAPSS2 mRNA levels and about 5-fold higher levels for SULT2A1 mRNA (Table S1). Condition 2 is basically fulfilled in any of the tissues where SULT2A1 is expressed: it is found strongly enriched in adrenal cortex, duodenum, liver, and small intestine (31, 32). In all these tissues, transcript abundance of SULT2A1 is higher than that of PAPS synthases (31) and another sulfotransferase, SULT1A1, is considerably co-expressed (31). In fact, cytoplasmic sulfotransferases are generally highly abundant, up to about 1% of total soluble protein (“cytosolic fraction”) of intestine tissue was reported to consist of the SULT enzymes SULT1A1/1A3, -1B1, -2A1, and -1E1 (32); corresponding to 20–30 μm sulfotransferase proteins. This means that there are many more PAPS-utilizing enzymes than PAPS synthases and sulfotransferases may even outnumber the PAPS cofactor itself (33), making a transient and isoform-specific interaction highly relevant for a functioning sulfation pathway.

In the crowded and complex environment of the living cell, proteins tend to form higher-order, transient protein–protein interactions (34), mostly remaining unnoticed or at least very hard to study (35). These have been termed “quinary interactions” (34), originally linked to an apparent conservation of isoelectric points among homologous proteins (36). The two human PAPS synthases PAPSS1 and PAPSS2 show very different isoelectric points, 6.40 and 8.18, respectively (Table S4). Taking the pI of SULT2A1 (pI = 5.69) into account, a more acidic (lower) value than most of the other cytoplasmic SULTs (Table S4), a transient interaction between PAPSS2 and SULT2A1, driven mainly by electrostatic interactions, is in good agreement with what our MM-PBSA free energy calculations show (Fig. 6*B*). Counterintuitively, the PAPSS2 residues at the SULT2A1 interface are mainly conserved in PAPSS1 (Table S3). This suggests that the difference between PAPS synthase isoforms may be caused by residues outside the protein interface, possibly in the second or even third shell of the protein (37).

A striking feature of the PAPSS2 interface with SULT2A1 is its composite nature (Fig. 7, *A* and *B*). Within the PAPSS2 dimer, the N terminus of the distant subunit swaps over to form the SULT2A1-binding site together with residues from the proximal subunit, initially seen crystallographically for PAPSS1 (38). Then it was observed in protein dissociation/association studies using fluorescently labeled PAPS synthases, PAPSS2 adopts this conformation about 2.5-fold quicker than PAPSS1 (20). An N terminally-truncated PAPSS1 protein, however, does not show any different catalytic properties compared with WT (38); parts of the protein responsible for quinary protein interactions are obviously dispensable for the primary catalytic function, as previously described for other quinary interactions (34). This N-terminal peptide shows large displacement values within the r.m.s. fluctuations calculations over the MD simulation time (Table S3), indicating that its flexibility may play a role for the PAPSS–SULT2A1 interaction; these r.m.s. fluctuation values are also higher in PAPSS2 than in PAPSS1.

Analyzing RMSF data allows assessing the effect of the novel PAPSS2–SULT2A1 protein interaction on the PAPS synthase. Within dimeric PAPSS2, nearly all 61 residues at the SULT2A1 interface show lower r.m.s. fluctuation values in the proximity to the sulfotransferase, compared with the corresponding amino acid in the distant PAPS synthase subunit (Table S3), which can be interpreted as stabilizing the otherwise fragile protein (4). In the past, PAPS synthases have been purported to have vastly differing specific activities (19), which actually was only a 5-fold difference in *k*_cat_/*K_m_* values when treating bi-functional PAPS synthases as Michaelis-Menten enzymes. For APS kinase, which catalyzes the rate-limiting step in overall PAPS biosynthesis, we did not observe this difference previously (20) nor in the present study (Fig. 2*A*). Using fluorescently labeled APS, we determined binding affinity of this nucleotide to PAPS synthases that theoretically have six APS-binding sites per protein dimer. This apparent *K_D_* of APS is somewhat larger for PAPSS2 (meaning APS binds less tightly) than for PAPSS1. APS was described as a modulator of PAPS synthase function (6) and more of it would be needed for intracellular stabilization of PAPSS2; making stabilization of PAPSS2 by a quinary interaction a likely alternative.

Soluble sulfotransferases are believed to form dimers via an unusually small binding interface spanning only 10 residues, known as the KTVE motif (39), whereas dimer formation of Golgi-localized sulfotransferases is mainly guided by their stem regions (40, 41). Notably, dimer formation was reported to be beneficial for sulfotransferase protein stability (42). This presumably “sticky” motif did not interfere with our docking experiments; it was never found to be enriched at the PAPSS2–SULT2A1 protein interface. In fact, the KTVE motif merges into the PAPS-binding loop on the other side of the sulfotransferase molecule (43). Comparing trajectories of MD simulations has been used in the past to approximate protein stability (44, 45). The SULT2A1 MD trajectory for the PAPSS2–SULT2A1 simulation certainly differs from the corresponding PAPSS1 complex; suggesting the SULT2A1 molecule reaching a stable state sooner (<1 ns) in the presence of PAPSS2 than PAPSS1 (Fig. S2). Furthermore, the average r.m.s. deviation of SULT2A1 stays at around 3 Å in the presence of PAPSS2, but further increases with PAPSS1. The presence of PAPSS2 seemingly stabilizes the SULT2A1 protein. Sulfotransferases feature three major substrate-binding “loops” based on early structural characterization and sequence alignments (46, 47). For SULT2A1, these would include Asp^62^–Arg^74^, Ser^80^–Gly^83^, and Glu^207^–Ser^251^; however, most of these residues are within well-defined secondary structure elements. In the three SULT2A1 crystal structures that we used for the present study (PDB codes 1EFH, 3F3Y, and 4IFB), a better description of the substrate-binding site are the loops Pro^14^–Ser^20^, Glu^79^–Ile^82^, and Asn^136^–Lys^144^ as well as the extended Tyr^231^–Gln^244^ loop that covers the binding pocket (43). These substrate-binding loops harbor all PAPSS2-contacting amino acids, except Glu^73^ and Glu^89^. Glu^89^ is located on the other end of a short helix containing the Glu^79^–Ile^82^ motif; Glu^73^ is part of a helical loop spanning Ile^71^–Arg^74^ and just 5 Å apart from the substrate lithocholic acid in the PDB 3F3Y structure. Being located in substrate-binding loops, all these residues are at least 15 Å apart from the 5′-phosphorous atom of the 3′-phosphoadenosine-5′-phosphate (PAP) cofactor. Thus, it seems unlikely that the PAPSS2–SULT2A1 interaction facilitates cofactor transfer. Instead, allosteric activation as recently described for SULT1A1 (48) and/or protein stabilization may be the mechanisms responsible for increased support of DHEA sulfation.

Recent amino acid exchanges within the substrate-binding loops of SULT2A1 may make the PAPSS2–SULT2A1 interaction specific to hominid primates only (Fig. 7) and this coincides with significantly higher DHEAS/DHEA rates in the circulation in anthropoid primates (28), contrasted to other primates and other mammals (29, 30). Adaptive changes in anthropoid proteomes have also been described for other genes (49). One of these is another sulfotransferase, SULT1A3, with a glutamic acid Glu^146^ within the substrate-binding site making it a preferential catecholamine-sulfating enzyme (50). SULT1A3 (and a duplicated gene named *SULT1A4* encoding the same protein) is the only sulfotransferase to have an acidic amino acid in this position. SULT1A3/4 genes have only been found in higher primates (New World monkeys, Old World monkeys, great apes, and humans) so far (32, 51), suggesting a strong evolutionary drive to develop this particular capacity specifically in higher primates. Similar to this finding, the current study about hominoid-specific facilitated DHEA sulfation may be relevant for the validity of animal models of steroid sulfation with significant implications for drug development relying on the predictive quality of animal models.

In conclusion, we have elucidated the mechanistic basis for the selective requirement for PAPSS2 in providing the sulfation cofactor PAPS to the DHEA sulfotransferase SULT2A1. This preference is explained by an isoform-specific, transient protein interaction of PAPSS2 and SULT2A1. SULT2A1 mainly interacts with PAPSS2 via residues within its nonconserved substrate-binding loops. Our analyses with PAPSS2 and the closely related SULT2B1 sulfotransferase confirm that this interaction is specific to SULT2A1. Isoform specificity on the side of PAPS synthases arises from nonconserved second- and third-shell residues causing differences in protein flexibility and electrostatics. The PAPSS2–SULT2A1 interaction stabilizes the interaction partners and may even allosterically activate them. The current findings show a novel regulatory mechanism within sulfation pathways and deepen our understanding of PAPS synthase biochemistry; they may help to better understand clinically observed *PAPSS2* mutations and even open new avenues to develop novel therapeutic targets.

## Materials and methods

### Cell culture

Adrenal NCI-H295R1 cells (kindly provided by Enzo Lalli, Nice, France) were grown in Dulbecco's modified Eagle's medium/F-12 (Gibco, Thermo Fisher, Waltham, MA) supplemented with 2.5% Nu-Serum, 1% ITS+ premix, and 1% penicillin/streptomycin at 37 °C and 5% CO_2_. HEK293 cells were propagated in minimal essential medium (Sigma) with 10% FCS (PAA, GE Healthcare) and 1% penicillin/streptomycin (PAA, GE Healthcare). HepG2 cells were maintained in RPMI (Invitrogen, Karlsruhe, Germany) with 10% FBS (Gibco, Thermo Fisher) and 1% antibiotic-antimycotic (Gibco, Thermo Fisher) in a humidified 5% CO_2_ atmosphere at 37 °C as recommended by the American Type Culture Collection (ATCC). All cells were verified to be mycoplasma-negative by PCR in regular intervals.

### Knockdown by siRNA in adrenal NCI-H295R1 cells

Adrenal NCI-H295R1 cells were transfected with the following siRNA oligonucleotides (target (mRNA position) RNA sequence (sense strand 5′ to 3′)): PAPSS1 (309) CCU GGU UUG UCA UGG UAU U; (419) GCA UCG CAG AAG UUG CUA A; (1380) GCA GGA UAC CCA UAA GCA A; PAPSS2, (612) CCA GCU UUA UUU CUC CAU U; (899) GCA GAA CAU UGU ACC CUA U; (1180) CCG UCU CUG CAG AGG AUA A; SULT2A1 (169) GCA UAG CUU UCC CUA CUA U; (622) GGU CAU GGU UUG ACC ACA U; (763) CCG AAG AAC UGA ACU UAA U; control, GCC ACG UAA GAU GAG UCA A; using the Viromer Blue transfection reagent (Lipocalyx, Halle/Saale, Germany) according to the manufacturer's instructions. Knockdown efficiency was checked by quantitative PCR using the following exon-spanning gene expression assays, all TaqMan probes were labeled with 6-carboxyfluorescein (FAM) (Life Technologies, Thermo Fisher): Hs00234219_m1 (SULT2A1); Hs00968937_m1 (PAPSS1); and Hs00989921_m1 (PAPSS2). Expression levels were normalized to 18S rRNA (HS99999901_s1). Protein expression was probed by Western blotting using the polyclonal rabbit antibody ab38416 (Abcam, Cambridge, UK) or the monoclonal mouse antibody SAB1100881 (Sigma) for SULT2A1, the mAb ab56398 (Abcam, Cambridge, UK) against PAPSS1 and the mAb ab56393 (Abcam) for PAPSS2. Equal loading was confirmed with horseradish peroxidase-linked mAb ab20272 (Abcam) against β-actin. ECL (Millipore, Watford, UK) or anti-mouse ReadyTector solution (CandorBioscience, Wangen im Allgäu, Germany) were used for detection.

### Overexpression of PAPS synthase variants in HEK293 cells

PAPS synthase protein variants with preferred nuclear or cytoplasmic localization have been described previously (18). Mutating K9A,K10A in PAPSS1 or K6A,K8A in PAPSS2 disrupts a conserved nuclear localization signal and results in preferential cytoplasmic localization. Changing R111A,R112A in PAPSS1 or R101A,R102A in PAPSS2, on the other hand, inactivates a motif with nuclear export signal activity, resulting in pronounced nuclear accumulation (18). Coding sequences for all these protein variants without stop codons were NheI/BamHI inserted in the eukaryotic expression vector pEGFP-N1 with a C-terminal EGFP fusion. HEK293 cells were transiently co-transfected with these plasmids and a SULT2A1 expression vector (14) using XtremeGene HP (Sigma) according to the manufacturer's protocol.

### Enzymatic assays

The functionality of the DHEA sulfation pathway was assessed by DHEA sulfation assays. NCI-H295R1 cells were incubated with 250 nm DHEA and 0.2 μCi of [^3^H]DHEA for 2 h at 37 °C; all assays were performed in triplicate. Steroids were extracted as previously described (13, 14), analyzed on a LablogicAR2000 bioscanner, and identified by referring to simultaneously run labeled steroid standards. Sulfation activity after transfection of scrambled control oligonucleotides was set to 100% activity. APS kinase activity was measured according to the STRENDA convention as previously described (4, 20). Briefly, ADP produced in the APS kinase-catalyzed reaction was used by pyruvate kinase to convert phosphoenolpyruvate to pyruvate. This is then converted by lactate dehydrogenase to lactate. The concurrent conversion of NADH to NAD^+^ is followed spectrophotometrically at 340 nm. APS kinase assays were carried out at 20 mm Tris-HCl, pH 7.3, 100 mm KCl, 5 mm DTT, 2.5 mm ATP, 15 μm APS, 10 mm MgCl_2_, 17.5/25 units of LDH/protein kinase mix, 2 units of nuclease P1, 0.8 mm phosphoenolpyruvate, 0.3 mm NADH, 30 μg/ml of PAPS. For APS ligand binding studies, mant-APS was obtained from Jena Bioscience (Jena, Germany) where an *N*-methylanthraniloyl fluorophore was esterified to the (2′,3′)-hydroxyl of the ribose moiety. Mant-APS was at a concentration of 1 μm for protein binding studies; for back-titration with label-free APS, 50 μm protein was added.

### Immunofluorescence

For immunofluorescence staining, human HepG2 cells were seeded in 35-mm glass bottom dishes (MatTek, Ashland, MA). Cells were fixed with 4% Histofix (Carl Roth, Karlsruhe, Germany) for 20 min at room temperature, followed by blocking and permeabilization with PBS containing 5% normal serum (Dako, Glostrup, Denmark) and 0.3% Triton X-100 (AppliChem, Darmstadt, Germany). Immunostaining was performed overnight at 4 °C with primary antibodies specific for PAPSS1 or PAPSS2 (both Abcam, Cambridge, UK) and SULT2A1 (Sigma) diluted 1:200 in PBS containing 1% BSA (Carl Roth, Karlsruhe, Germany) and 0.3% Triton X-100. Following several washing steps, secondary antibodies labeled with Alexa Fluor 488 and Alexa Fluor 568 (Life Technologies, Thermo Fisher) were incubated for 1 h at room temperature. DNA was stained with Hoechst 33342 (AppliChem, Darmstadt, Germany) in PBS for 15 min at room temperature. Images (Fig. S1*B*) were taken with a Leica SP8 confocal microscope equipped with a HCX PL Apo CS 63.0 × 1.20 water UV objective, a sensitive hybrid detector and Diode 405, Argon and DPSS561 lasers (Leica, Wetzlar, Germany).

### Proximity ligation assays

Interactions of sulfation pathway proteins were tested for by PLA technology. Human HepG2 cells were grown, fixed, blocked, and permeabilized as described above. Cells were then incubated overnight at 4 °C with primary antibodies specific for PAPSS1 or PAPSS2 (both from Abcam) and SULT2A1 (Sigma) diluted 1:200 in PBS containing 1% BSA (Carl Roth, Karlsruhe, Germany) and 0.3% Triton X-100. PLA was conducted using the Duolink In Situ PLA probes and detection reagents (Sigma), following the instructions of the manufacturer. DNA was stained with Hoechst 33342 (AppliChem, Darmstadt, Germany) in PBS for 15 min at room temperature; entire cells were stained with HCS CellMask Deep Red Stain (Life Technologies, Thermo Fisher) in PBS for 15 min at room temperature. Images were taken with a Leica SP8 confocal microscope equipped with a HCX PL Apo CS 63.0 × 1.20 water UV objective, a sensitive hybrid detector and Diode 405, Argon and DPSS561 lasers, and further analyzed with CellProfiler (52, 53). We noticed that the overall intensity of the PLA signal inversely correlated with cell density: when grown more densely, lower PLA signal intensities were observed. However, cell density did not affect foci number.

### Structural analysis and molecular docking

Full all-atom models of PAPSS1 and PAPSS2 were built with the MMMserver (54) using the crystal structures of both the isolated kinase domain and an APS complex of full-length PAPSS1 (PDB 2OFX and 1XNJ, respectively). Homology building also for PAPSS1 ensured that we used comparable structures of PAPSS1 and PAPSS2 coherently throughout this study. To elucidate the binding sites and investigate the mode of binding, these optimized models of both PAPSS1 and PAPSS2 were docked to three different SULT2A1 structures (PDB codes 3F3Y, 4IFB, and 1EFH) and to SULT2B1 (PDB code 1Q1Q) by the rigid-body protein–protein docking software ClusPro. PAPS synthase and sulfotransferase structures were submitted as receptor and ligand, respectively, to the ClusPro protein–protein docking server using default settings (24). The top 1000 lowest energy-docking poses of aforementioned complexes are grouped into 30 clusters and the lowest energy poses of each cluster form the final 30 docking poses. Docking results were scored using the standard ClusPro “balanced docking score,” where all electrostatic, hydrophobic, and van der Waals + Elec coefficients are taken into account. All 30 ClusPro poses where further filtered by populating the top amino acid contacts within all complex structures. To analyze the involvement of each amino acid in the protein–protein interaction interfaces, we calculated the prevalence of interface interactions for the receptor PAPSS residues with contacts made to the ligand SULTs in all dockings.

For each PAPS synthase/sulfotransferase system, MD simulations were run as the following. The protein complex was inserted in a dodecahedric box of TIP3P water molecules ensuring a minimum distance to the box edges of 10 Å. The proper amount of Na^+^ and Cl^−^ ions was added to reach an ionic concentration of 150 mm and ensure final neutral systems. A steepest-descent minimization was applied to relax the solvent molecules around the solute. The equilibration was performed in two steps: the system was at first thermalized up to 300 K coupling the protein and the solvent to a V-rescale thermostat (τt = 0.1 ps) in the canonical ensemble (NVT). Then, we switched to the NPT statistical ensemble, performing 100 ps of MD at 300 K, coupling the system with a Parrinello-Rahman barostat (τp = 2 ps). After this initial phase, the system was submitted for production MD simulations. Production runs were carried out in the NPT (*p* = 1 bar, T = 300 K) statistical ensemble. All bonds were constrained with LINCS (55), allowing to use a time step set of 2 fs. Periodic boundary conditions were applied to the systems in all directions. The PME method was used to evaluate long-range electrostatic interactions (PME order = 4, Fourier spacing = 0.12), and a cutoff of 10 Å was used to account for the van der Waals interactions. Coordinates of the systems were collected every 2 ps. All MD simulations were carried out with GROMACS-5 using the Gromos 53A6 force field on GPU/CPU machines. The length of the MD simulations was 20 ns, and standard MD were used for MM-PBSA and for all analysis. Binding free energy were calculated using the GROMACS tool g_mmpbsa (56).

### Data analysis

Enzyme kinetics and titration data were analyzed and visualized using GraphPad Prism. Densitometric analysis of bands on Western blots was carried out with GelAnalyzer. Visualization of protein structures and structural models was done in PyMol, VMD, and YASARA. Normality of any data were checked for by visual inspection of histogram plots as well as with Ryan-Joiner and D'Agostino-Person tests. Pairwise and multiple comparisons of normally distributed data were done with two-tailed unpaired *t* tests and one-way ANOVA as well as post hoc Bonferroni tests, respectively. All statistical analyses were carried out with the Analysis ToolPak-VBA (Microsoft) or Minitab 17.

## Author contributions

J. W. M. and W. A. conceptualization; J. W. M., S. K. K., and W. A. resources; J. W. M., J. I., T. F. G., C. V., R. H., and J. v. d. B. data curation; J. W. M., J. I., T. F. G., C. V., R. H., J. v. d. B., V. D., S. K. K., E. R., and W. A. formal analysis; J. W. M., V. D., S. K. K., E. R., and W. A. supervision; J. W. M. and W. A. funding acquisition; J. W. M., J. I., R. H., and J. v. d. B. investigation; J. W. M., T. F. G., C. V., and S. K. K. visualization; J. W. M., J. I., C. V., J. v. d. B., S. K. K., and E. R. methodology; J. W. M. writing-original draft; J. W. M. and W. A. project administration; J. W. M., J. I., T. F. G., C. V., R. H., J. v. d. B., V. D., S. K. K., E. R., and W. A. writing-review and editing; T. F. G. software; C. V., J. v. d. B., V. D., S. K. K., and E. R. validation.

## Supplementary Material

Supporting Information

## Footnotes

2 The abbreviations used are:

PAPS: 3′-phosphoadenosine-5′-phosphosulfate
APS: adenosine 5′-phosphosulfate
DHEA: dehydroepiandrosterone
DHEAS: dehydroepiandrosterone-3-sulfate
EGFP: enhanced green fluorescent protein
MD: molecular dynamics
MM-PBSA: molecular mechanics-Poisson-Boltzmann surface area
PAP: adenosine 3′-phospho-5′-phosphate
PAPSS: PAPS synthase
PLA: proximity ligation assay
r.m.s.: root mean square
SULT2A1: (DHEA) sulfotransferase 2A1
PDB: Protein Data Bank
ANOVA: analysis of variance.
