# Supplementary material for: Human DHEA sulfation requires direct interaction between PAPS synthase 2 and DHEA sulfotransferase SULT2A1
Source: J Biol Chem. 2018 May 9;293(25):9724–35. doi: 10.1074/jbc.RA118.002248 (PMC6016456; doi:10.1074/jbc.RA118.002248)
Supplement: Supporting Information [file supp_293_25_9724__index.html]

Human DHEA sulfation requires direct interaction between PAPS synthase 2 and DHEA sulfotransferase SULT2A1 — DHEA sulfation and PAPSS2–SULT2A1 interaction — Supporting Information 

# Human DHEA sulfation requires direct interaction between PAPS synthase 2 and DHEA sulfotransferase SULT2A1

## Supporting Information

- Supp. Figures 1 & 2 - Supp. Figures 1 & 2
