# Supplementary material for: Human DHEA sulfation requires direct interaction between PAPS synthase 2 and DHEA sulfotransferase SULT2A1
Source: J Biol Chem. 2018 May 9;293(25):9724–35. doi: 10.1074/jbc.RA118.002248 (PMC6016456; doi:10.1074/jbc.RA118.002248)
Supplement: Supporting Information [file supp_RA118.002248_135768_1_supp_128342_p7svrj.pdf]

SUPPORTING INFORMATION

for the article

Human DHEA sulfation requires direct interaction between PAPS synthase 2 and DHEA sulfotransferase SULT2A1

by

Jonathan W Mueller, Jan Idkowiak, Tarsis F. Gesteira, Cecilia Vallet, Rebecca Hardman, Johannes van den Boom, Vivek Dhir, Shirley K. Knauer, Edina Rosta, Wiebke Arlt

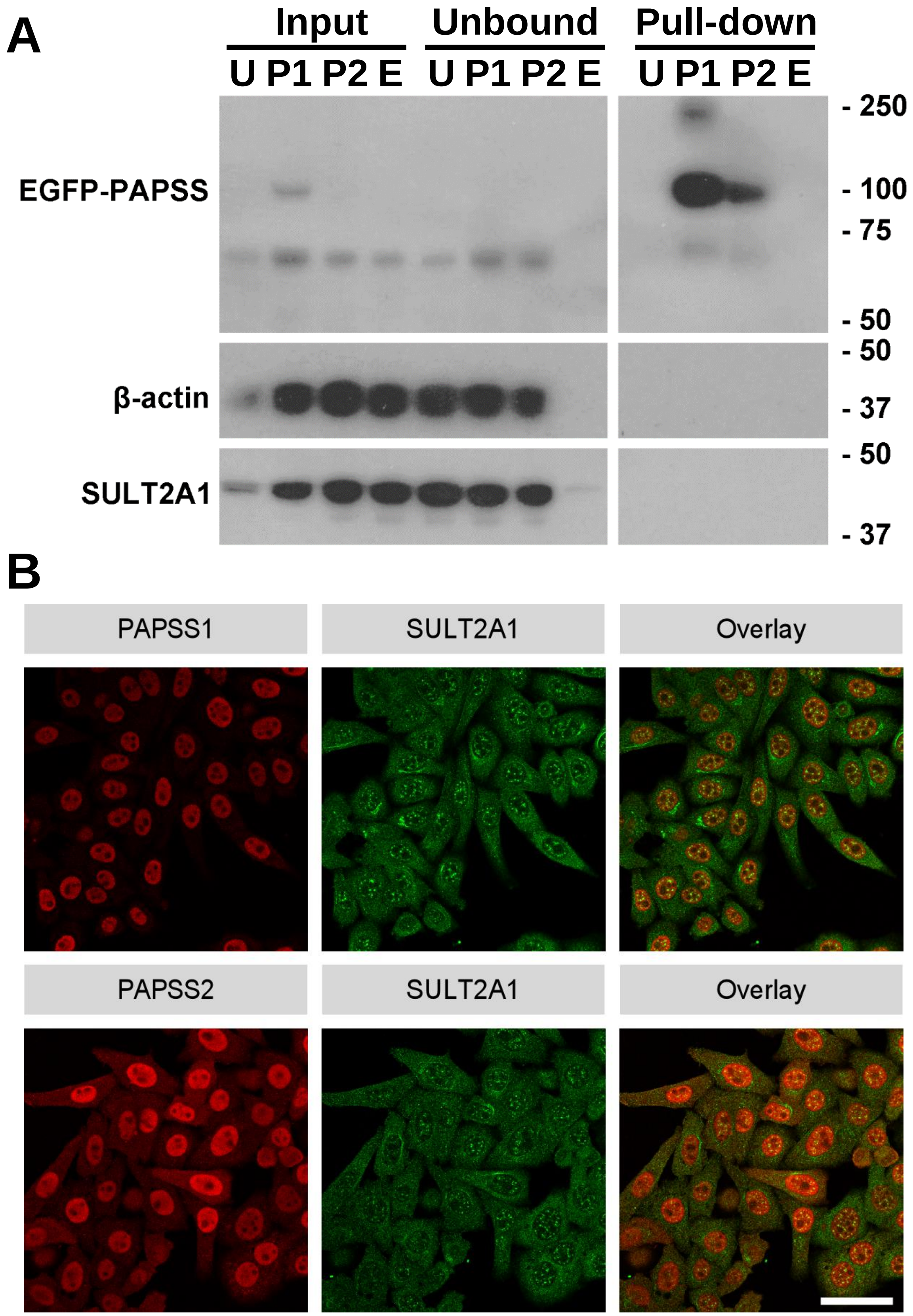

Supporting Figure 1: GFP-trap-mediated PAPS synthase pull down and immuno-histochemical staining of sulfation pathway proteins

A, EGFP-tagged PAPS synthases were over-expressed in NCI-H295R1 cells and pulled down from protein lysates using GFPtrap technology (Chromotek, Planegg-Martinsried, Germany). U, untreated; P1/P2, PAPS synthases 1/2; E, empty vector. Detection was with antibodies against EGFP, β-actin and human SULT2A1. A weak EGFP-PAPSS band is visible in the input, but very much more in the pull-down. Endogenous SULT2A1, clearly present in the input, does not co-elute together with PAPS synthases. **B**, mouse monoclonal antibodies for PAPSS1 or PAPSS2 were combined with a rabbit SULT2A1 polyclonal antibody for detection of endogenous proteins in a HepG2 cell line. Magnification, 600x.

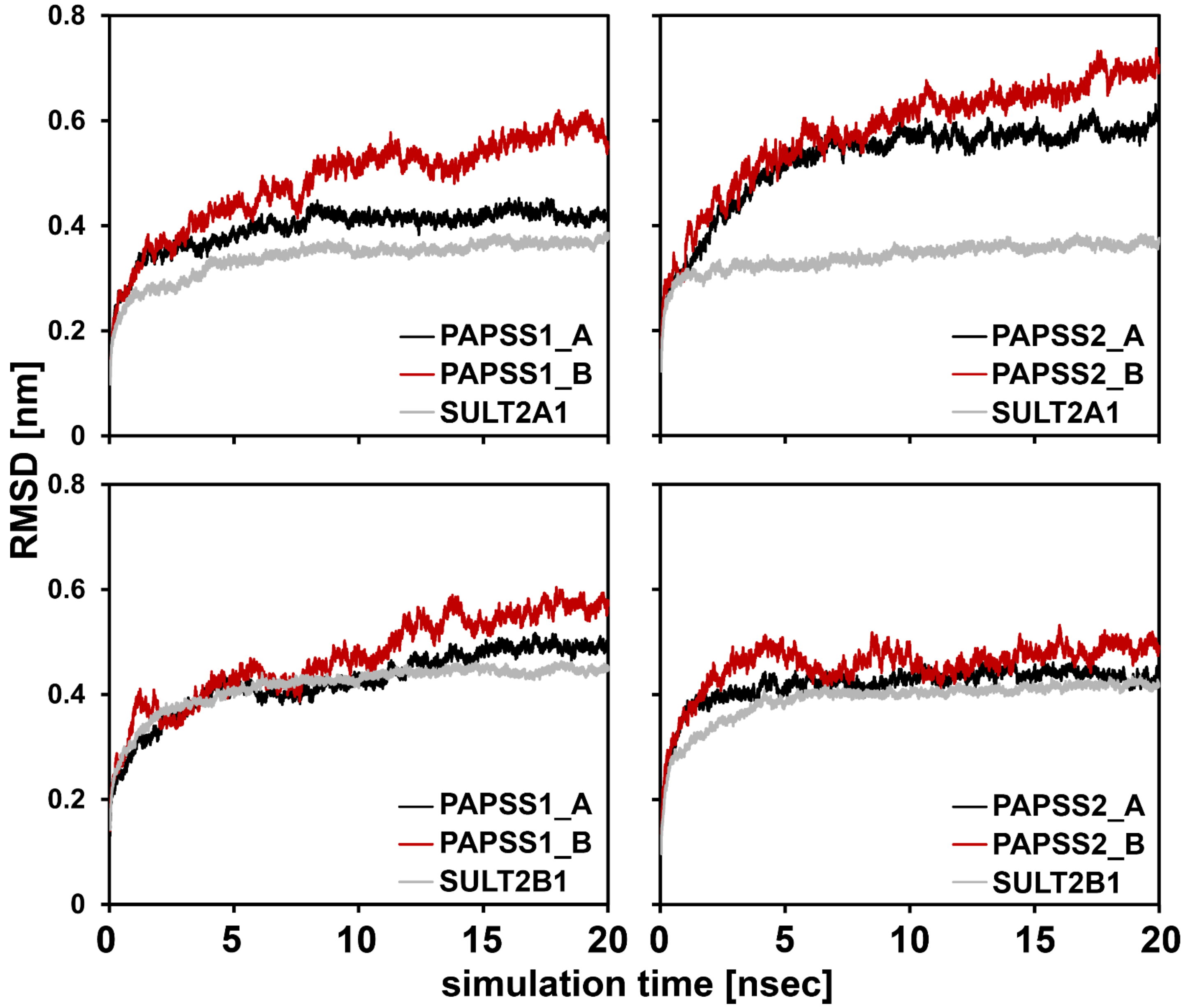

Supporting Figure 2: RMSD traces for PAPS synthase-sulfotransferase MD simulations
Trajectories of three MD simulations were averaged as described (Liou et al, PLoS One 9, e94178) and plotted over 20 ns simulation time. Black, the PAPS synthase subunit "A" proximal to the sulfotransferase; red, the PAPS synthase subunit "B" more distant to the sulfotransferase; gray, the sulfotransferase. All four systems stabilized at a local RMSD minimum during simulation time (hence, MM-PBSA calculations are possible). Notably, the "B" subunit, the one not engaged in SULT binding, displays consistently higher RMSD values.

Supporting Table 1: Real-time PCR data for NCI-H295R1 knockdown studies

$\Delta C_T \pm SD$	**siSULT2A1** (control)	**siPAPSS1** (control)	**siPAPSS2** (control)
SULT2A1	**15.84±0.87** (13.79±0.72)	**12.76±1.51** (12.90±1.60)	**13.08±0.48** (13.81±0.46)
PAPSS1	**16.72±0.19** (16.58±0.22)	**17.27±1.63** (15.25±1.48)	**15.25±0.74** (16.07±0.68)
PAPSS2	**15.10±0.54** (15.82±0.41)	**15.97±0.61** (15.43±0.44)	**17.56±0.43** (15.90±1.15)
At least five replicates were done for each measurement			

Supporting Table 2: DHEA conversion rates for PAPS synthase localization variants

PAPS synthase variant[a]	DHEA conversion rate[b] **[nmol DHEA/hour]**	p-value[c] (relative to WT)
empty vector	2.03 ± 0.13	
PAPSS1 wild type	13.87 ± 1.33	
PAPSS1 KK9,10AA	8.51 ± 0.83	< 0.05
PAPSS1 RR111,112AA	10.04 ± 0.67	< 0.05
PAPSS2 wild type	29.1 ± 1.95	
PAPSS2 KK6,8AA	26.9 ± 1.29	no difference to WT
PAPSS2 RR101,102AA	22.56 ± 1.22	< 0.01

[a] each PAPSS variant was co-transfected with SULT2A1
[b] all values expressed as the mean (± SEM) of triplicate assays from three independent experiments.
[c] p value from post-hoc Bonferroni tests after one-way ANOVA (p-value < 0.001)

Supporting Table 3: PAPS synthase residues at the SULT2A1 interface

PAPSS2			SULT2A1 distance**	RMSF data for PAPS synthases				PAPSS1	
AA-P2	mol	no-P2		P2_A	P2_B	P1_A	P1_B	AA-P1	no-P1
SER*	B	15	dist<5	0.510	0.492	0.347	0.508	ALA	25
THR	B	16	dist<7	0.397	0.442	0.337	0.427	THR	26
ASN	B	17	dist<3	0.444	0.414	0.290	0.419	ASN	27
VAL	B	18	dist<3	0.353	0.338	0.233	0.299	VAL	28
VAL*	B	19	dist<5	0.360	0.327	0.244	0.297	THR	29
TYR	B	20	dist<3	0.371	0.302	0.238	0.306	TYR	30
GLN	B	21	dist<3	0.336	0.302	0.233	0.254	GLN	31
ALA	B	22	dist<3	0.302	0.276	0.214	0.239	ALA	32
HIS	B	23	dist<5	0.296	0.272	0.197	0.238	HIS	33
HIS	B	24	dist<3	0.306	0.264	0.231	0.219	HIS	34
VAL	B	25	dist<5	0.242	0.233	0.176	0.169	VAL	35
SER	B	26	dist<5	0.245	0.225	0.179	0.170	SER	36
ARG	B	27	dist<3	0.259	0.220	0.195	0.214	ARG	37
ASN	B	28	dist<5	0.276	0.244	0.226	0.206	ASN	38
LYS	B	29	dist<5	0.243	0.248	0.229	0.193	LYS	39
ARG	B	30	dist<7	0.207	0.210	0.160	0.146	ARG	40
GLY	A	52	dist<7	0.171	0.256	0.155	0.198	GLY	62
ALA	A	53	dist<7	0.160	0.260	0.165	0.221	ALA	63
GLY	A	54	dist<5	0.153	0.252	0.145	0.201	GLY	64
LYS	A	55	dist<7	0.133	0.214	0.114	0.183	LYS	65
THR	A	56	dist<5	0.148	0.208	0.145	0.182	THR	66
THR	A	57	dist<3	0.155	0.233	0.138	0.187	THR	67
ILE	A	58	dist<7	0.133	0.209	0.107	0.167	VAL	68
PHE*	A	60	dist<5	0.164	0.212	0.175	0.149	MET	70
ALA	A	61	dist<7	0.135	0.194	0.096	0.147	ALA	71
GLU	A	64	dist<3	0.169	0.219	0.119	0.164	GLU	74
TYR	A	65	dist<7	0.174	0.230	0.107	0.182	TYR	75
ASP	A	79	dist<5	0.204	0.243	0.135	0.165	ASP	89
ASN	A	80	dist<7	0.229	0.235	0.139	0.158	ASN	90
VAL	A	81	dist<7	0.190	0.213	0.130	0.179	ILE	91
ARG	A	82	dist<5	0.179	0.250	0.148	0.202	ARG	92
HIS	A	83	dist<3	0.236	0.280	0.189	0.206	GLN	93
GLY	A	84	dist<7	0.243	0.260	0.153	0.187	GLY	94
ARG	A	87	dist<7	0.330	0.333	0.220	0.242	LYS	97
ASP	B	112	dist<7	0.187	0.187	0.138	0.160	ASP	122
SER	B	138	dist<7	0.250	0.239	0.201	0.242	GLY	148
ALA	B	139	dist<7	0.223	0.216	0.178	0.233	ALA	149
GLY	B	140	dist<7	0.215	0.207	0.189	0.217	SER	150
SER	A	157	dist<7	0.306	0.381	0.267	0.331	GLN	167

ARG	A	158	dist<5	0.265	0.368	0.297	0.294	ARG	168
ASP	A	159	dist<5	0.259	0.336	0.208	0.231	ASP	169
VAL	A	160	dist<5	0.286	0.370	0.262	0.223	VAL	170
LYS	A	161	dist<5	0.233	0.337	0.208	0.201	LYS	171
GLY	A	162	dist<5	0.255	0.345	0.221	0.215	GLY	172
LEU	A	163	dist<7	0.248	0.306	0.214	0.229	LEU	173
LYS	A	165	dist<3	0.364	0.412	0.244	0.265	LYS	175
ARG	A	166	dist<3	0.371	0.383	0.263	0.246	LYS	176
ARG	A	168	dist<7	0.350	0.414	0.277	0.311	ARG	178
GLU	A	171	dist<5	0.498	0.425	0.356	0.299	GLU	181
ILE	A	172	dist<7	0.347	0.331	0.240	0.239	ILE	182
THR	A	198	dist<7	0.227	0.302	0.205	0.266	ASP	208
VAL	A	199	dist<5	0.197	0.263	0.199	0.231	VAL	209
SER	A	200	dist<7	0.201	0.267	0.194	0.238	ASN	210
ARG	A	427	dist<7	0.208	0.281	0.173	0.193	LYS	437
ARG	A	428	dist<7	0.184	0.278	0.162	0.192	GLN	438
LEU	A	429	dist<7	0.132	0.211	0.109	0.155	LEU	439
LEU	A	430	dist<5	0.145	0.238	0.133	0.188	LEU	440
GLU	A	431	dist<3	0.188	0.287	0.187	0.223	GLU	441
ARG	A	432	dist<5	0.243	0.335	0.274	0.216	ARG	442
GLY	A	433	dist<5	0.168	0.260	0.112	0.206	GLY	443
TYR	A	434	dist<7	0.142	0.235	0.098	0.194	TYR	444

*these residues were highlighted as decisive between PAPSS1- and PAPSS2-like sulfate activating complexes before (van den Boom et al, J Biol Chem 287, 17645-17655).

**residues within 3, 5 or 7 Angstrom distance from SULT2A1 in an PAPSS2-SULT2A1 complex averaged over 15 ns simulation time

Supporting Table 4: Isoelectric points of DHEA sulfation pathway proteins

Organism	Enzyme	Identifier*	pI
Homo sapiens	PAPSS1	NM_005443	6.40
	PAPSS2	NM_004670	8.18
	SULT2A1	NM_003167	5.69
	SULT2B1	NM_004605	6.10
	SULT1A1	NM_001055	6.16
	SULT1B1	NM_014465	6.57
	SULT1E1	NM_005420	6.18

*NCBI RefSeq numbers are given
